# Gastric Emptying of Orange Juice With and Without Pulp: A Point-of-Care Ultrasound Study

**DOI:** 10.7759/cureus.30959

**Published:** 2022-11-01

**Authors:** Soleil Schutte, Sindhuja R Nimma, Cameron R Smith, Linda Le-Wendling

**Affiliations:** 1 Department of Anesthesiology, University of Florida, Gainesville, USA; 2 Department of Anesthesiology, Mayo Clinic, Jacksonville, USA

**Keywords:** aspiration risk, npo time, orange juice, gastric emptying, poc ultrasound

## Abstract

Purpose

The American Society of Anesthesiologists (ASA) preoperative fasting recommendations regarding fruit juice with pulp is unclear. In addition, it is debatable whether orange juice without pulp should be treated as a clear liquid. Our objective is to determine the gastric emptying time of orange juice with and without pulp.

Methods

This is an observational study of gastric emptying time using point-of-care ultrasound (POCUS). Thirty-five adult volunteers were enrolled in this study. Exclusion criteria included pregnancy, diabetes, body mass index > 40 kg/m^2^, previous lower esophageal or upper abdominal surgery, hiatal hernia, and upper gastrointestinal bleed. The study was carried out on three separate days for each volunteer. After fasting a minimum of 8 h, the volunteers were asked to drink 240 ml of water on day 1, orange juice without pulp on day 2, and orange juice with pulp on day 3. Gastric volumes were estimated using gastric antrum cross-sectional area at fasting state, and then 30, 60, 90 120, 180, and 240 min after drinking until the gastric volume returned to baseline.

Results

A gastric volume of 1.5 mL/kg was defined as a baseline. All subjects’ gastric volume returned to baseline 90 min after drinking water. More than 97% of the subjects who drank orange juice without pulp and 93.9% of the subjects who drank orange juice with pulp reached a gastric volume of less than 1.5 mL/kg after 2 h. All subjects’ gastric volume returned to baseline 3 h after drinking orange juice with pulp.

Conclusions

Orange juice without pulp can be treated as a clear liquid in a majority of patients who do not have conditions that would cause delayed gastric emptying. Orange juice with pulp required 3 h to empty.

## Introduction

Pulmonary aspiration of gastric contents is a major anesthetic-related complication. Although current guidelines do not guarantee any specific outcome, verifying patient compliance is prudent. Gastroenterologists first used gastric ultrasound to assess gastric motility in the 1980s [1]. In the last decade, POCUS was introduced as a noninvasive way to assess the risk of aspiration in preoperative patients because of its ability to allow for the assessment of gastric contents and to differentiate an empty stomach from one with liquid/solid contents [2-5].

Preoperative fasting guidelines from the American Society of Anesthesiologists (ASA) recommend a minimum fasting period of 2 h after clear liquids, which include water and fruit juices without pulp [6]. However, these guidelines do not specify which juices can be considered clear liquids. Early gastric emptying studies for clear liquids compared to water and apple juice, with limited evidence on the emptying times of orange juice. Many physicians delay surgery as they do not consider orange juice a clear liquid because it is not translucent [7]. There is also no clear fasting guideline for fruit juice with pulp. Some clinicians may consider 4 h enough because it is not as fatty as breast milk, whereas others may demand 6 or 8 h because of its solid components. To help improve understanding of this issue, we designed a study using gastric ultrasound to evaluate gastric emptying times of orange juice with and without pulp.

## Materials and methods

The University of Florida Institutional Review Board approved this study (IRB201802805). After obtaining written informed consent, we recruited 35 adult volunteers to participate. Exclusion criteria included pregnancy, diabetes, body mass index > 40 kg/m^2^, previous lower esophageal or upper abdominal surgery, hiatal hernia, and upper gastrointestinal bleed.

Each subject was asked to fast for 8 h overnight on three separate days. Baseline fasting gastric antrum cross-sectional area (CSA) in the supine and right lateral decubitus (RLD) position was obtained by using the imaging techniques described by Perlas et al. [2,3]. Briefly, a curvilinear probe (Sonosite C60xp/2-5 MHz; FUJIFILM Sonosite, Bothell, WA) was used to first identify the aorta in the cross-sectional view, just inferior and to the right of the xiphoid process. Once the aorta was centered on the screen, the ultrasound probe was rotated 90° to obtain a sagittal view where the gastric antrum was identified adjacent to the liver in the same sagittal plane as the aorta. The CSA of the antrum, excluding the serosa, was measured using the trace function.

Each subject then drank 240 mL of fluid as follows: water on test day 1, orange juice without pulp on test day 2, and orange juice with pulp on test day 3. Subsequently, gastric antrum CSAs in the supine and RLD position were obtained at 30, 60, 90, 120, 180, and 240 min after fluid intake until the subject’s estimated gastric volume returned to baseline. The scanners were not blinded to the fluids taken by subjects. The volume of gastric contents was estimated by using the following model proposed by Perlas et al. [8].

Volume (mL) = 27 + 14.6 × RLD-CSA - 1.28 × age 

**Figure 1:**
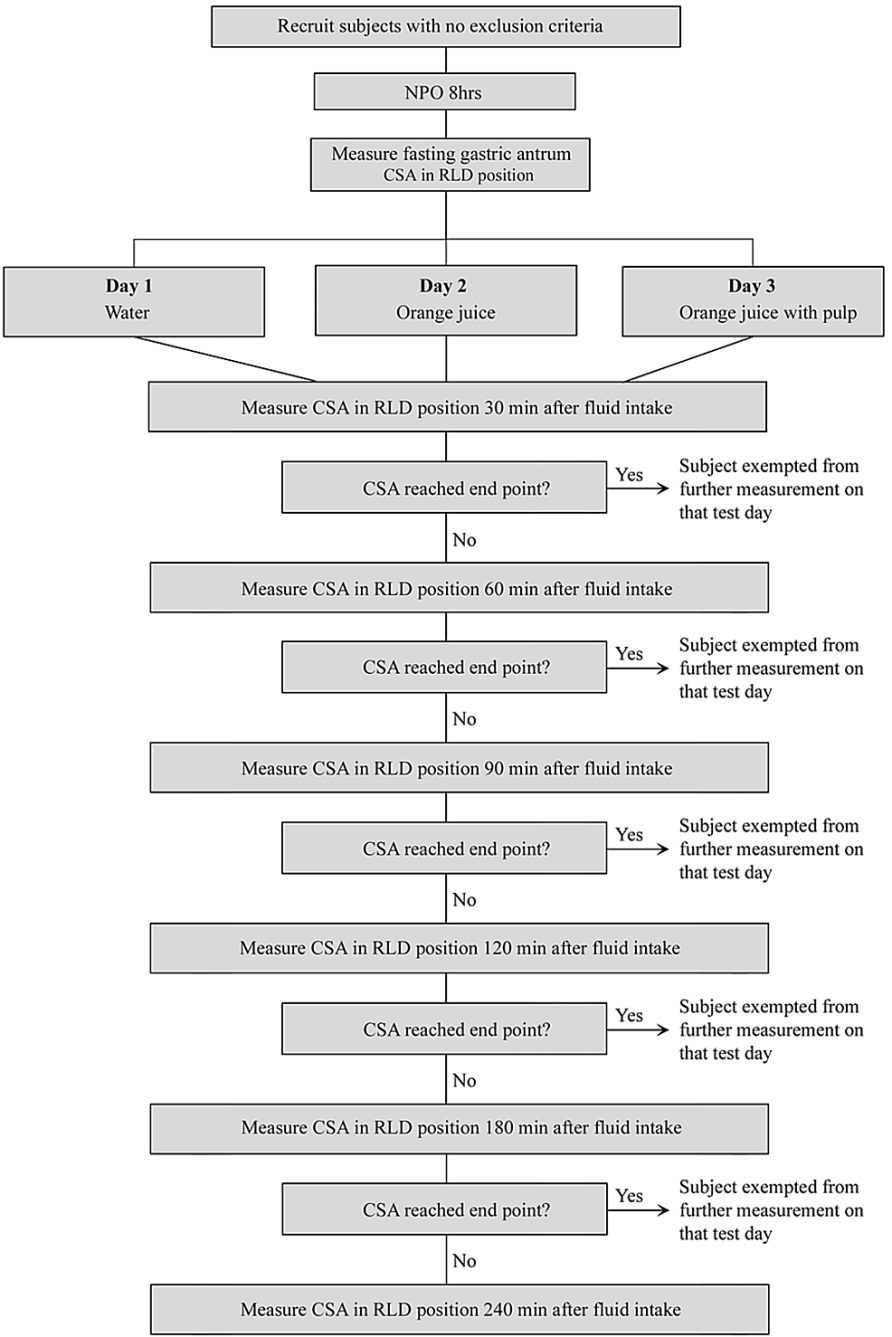
Experiment design.

Baseline gastric secretions are common during fasting, and a gastric volume of less than 1.5 mL/kg or 100 mL for an average-sized adult was generally considered a low risk for aspiration [8-10]. A gastric volume of 100 mL correlated to a CSA of 5 or 7 cm^2^ in the RLD position, depending on age, in our study subjects [8]. To simplify data collection, a CSA of either 5 or 7 cm^2^ in the RLD position, depending on the subject’s age, was used as an endpoint on each test day (Figure 1).

## Results

The study consisted of 27 male and eight female subjects. Five subjects had a history of gastroesophageal reflux disease but no other significant comorbidities. One of the subjects opted out of the study after test day 1 due to issues unrelated to the study. Demographic information is provided in Table 1.

**Table 1:** Demographic information

	Range	Mean ± SD
Age (yrs)	22–48	33.1 ± 6
Height (cm)	157–191	174.8 ± 9.8
Weight (kg)	54–102	76.7 ± 13.9
BMI (kg/m^2^)	19.0–38.4	25.1 ± 4.1

We successfully located the gastric antrum in the supine and RLD positions by using the ultrasound technique as referenced above in all 35 subjects. The fasting CSA in the supine position and the RLD position were measured and listed in Table 2. The estimated fasting gastric volume was calculated and provided in Table 2. Using the limit of 1.5 mL/kg, all subjects were considered at low risk for aspiration in the fasting state per the gastric ultrasound examination. After ingestion of water, all of the subjects were considered at low risk of aspiration at 90 min.

**Table 2:** Baseline gastric antrum CSA and volume

	Range	Mean ± SD
CSA-supine (cm^2^)	1.3 – 7.0	3.0 ± 1.3
CSA-RLD (cm^2^)	1.1 – 10.0	4.2 ± 2.1
Volume (mL)	3 – 132	48 ± 30

The sonographic images of orange juice without pulp correlated with those of a clear liquid described as hypoechoic appearing content. After ingestion of orange juice without pulp, 97.1% of the subjects were considered at low risk at 120 min. The only subject who was deemed at increased risk for aspiration at 120 min had an estimated gastric volume of 122 mL. He weighed 75 kg with a calculated baseline gastric volume of 113 mL.

**Figure 2:**
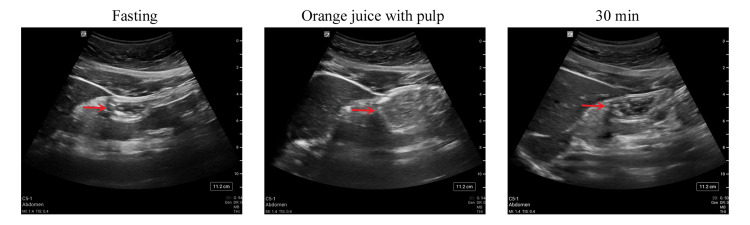
Ultrasonographic images of the gastric antrium Ultrasonographic images of the gastric atrium, as indicated by the red arrow, at fasting state, immediately after drinking orange juice with pulp, and 30 min afterward.

The sonographic images of orange juice with pulp were more consistent with those of liquid than those of solid (Figure 2), though the content is more hyperechoic than that of a clear liquid. After ingestion of orange juice with pulp, 93.9 % of the subjects were considered at low risk at 120 min and all were a low risk at 180 min.

## Discussion

In the absence of a history of gastroparesis, 97.1% of subjects who had consumed 240 mL of orange juice without pulp and 93.9% of subjects who consumed 240 mL of orange juice with pulp were determined to have gastric volumes of less than 1.5 mL/kg two hours after ingestion. Orange juice with pulp was emptied from the stomach of all participants within 3 h after ingestion, 1 h earlier than the 4-h fasting time for breast milk.

The gastric volume that is considered a minimal risk for aspiration is unknown. From studies measuring baseline gastric secretion volume, gastric volumes up to 1.5 mL/kg were common, considered normal, and used by Perlas et al. to stratify the risk of aspiration as minimal [8-10]. The fasting gastric volume of the subjects in this study were all within the 1.5 mL/kg limit. Based on this threshold, the addition of pulp prolonged gastric emptying in only one subject compared to orange juice without pulp.

The baseline gastric volume that was used for aspiration stratification was an estimate, not a gold standard. The sensitivity and specificity of this limit are unknown. Perlas et al. proposed another risk stratification method using a universal threshold of 100 mL to stratify the risk of aspiration. It assists clinicians to stratify the risk of aspiration without consideration of the patient’s weight [8]. However, it is more conservative for patients who are heavier than 66 kg compared to the 1.5 mL/kg weight-based standard. Based on this more conservative threshold, only 81.8% of the OJ with pulp group were considered low risk for aspiration at two hours. In the OJ group without pulp, 97.1% reached a volume of less than 100 mL by two hours. The sensitivity and specificity of either of these two cut-offs for predicting the risk of aspiration are unknown.

POCUS provides a useful way to evaluate the risk of aspiration in the perioperative setting, especially when the patient’s fasting status is unknown, the patient has gastroparesis, or when guidelines are ambiguous. However, interpreting the data can be challenging. Regardless of the gastric volume, the presence of solid components (hyperechoic heterogenous appearance of the contents) in the gastric antrum is considered to indicate a high risk for aspiration [2]. The mathematical model by Perlas et al. was validated for clear liquid only. A more recent study showed that it underestimated the volume of Nutricia Nutridrink, a fat-containing feeding drink [11]. Since the orange juice with pulp does not contain fat and the sonographic images were more consistent with those of clear liquid as time went by, we opted to use the mathematical model by Perlas et al. to estimate gastric volume. However, we cannot rule out that the gastric volume of orange juice with pulp was underestimated in this study.

Limitations of the study include the accuracy of the gastric volume estimate. Peristalsis of the stomach added variations to gastric antrum measurements and subsequent gastric volume estimation. Data collection and interpretation can also vary based on the technique and the experience of the individuals involved in both image acquisition and interpretation, patient positioning, and the type of food ingested, which can affect decision-making. In addition, the reliability and validity of using gastric ultrasound to estimate the risk of aspiration require further study. Certain pathology such as severe gastroesophageal reflux disease, diabetes, opioid use, poorly controlled pain, gastrointestinal dysmotility, previous gastrointestinal surgery, and end-stage renal disease may also affect gastric emptying [12,13], and our generally healthy population may not be generalizable in these situations.

## Conclusions

Orange juice without pulp was cleared from the stomach within two hours in all but one subject whose gastric volume was marginally higher than 1.5 mL/kg at two hours. We feel orange juice can be treated as a clear liquid in patients who do not have conditions that would cause delayed gastric emptying. Orange juice with pulp was cleared in 94% of the patients in two hours and emptied in all of the subjects in three hours.
